# Reconsidering the usefulness of using long-acting injectable buprenorphine as a tapering tool: a case report of delayed withdrawal months after last dose

**DOI:** 10.3389/fpsyt.2026.1863924

**Published:** 2026-07-06

**Authors:** Kareem Woods, Sophia Graham, Steven Dobscha, Christopher Blazes

**Affiliations:** 1 United States Department of Veterans Affairs, VA Portland Health Care System, Veterans Health Administration, Portland, OR, United States; 2 Oregon Health & Science University, Portland, OR, United States; 3 VA Portland Health Care System, Portland, OR, United States

**Keywords:** brixadi, buprenorphine, case report, delayed withdrawal, kappa opioid receptor, long-acting injectable, naltrexone, opioid use disorder

## Abstract

**Background:**

Long-acting injectable buprenorphine (LAIB) has generated enthusiasm as a potential tapering strategy for patients seeking discontinuation from buprenorphine therapy. The rationale is pharmacokinetic: gradual decline in plasma concentrations over months may blunt or prevent withdrawal. Yet protocols, clinical risks, and the characteristics of delayed withdrawal during LAIB tapers are poorly described.

**Case:**

We report a 45-year-old man who developed somatic withdrawal symptoms four months after receiving a single 200 mg dose of LAIB (Sublocade) as a tapering tool following years of stability on sublingual buprenorphine. Administration of 12.5 mg naltrexone precipitated a clinically significant worsening of withdrawal, supporting ongoing μ-opioid receptor agonism at month four. Symptoms were attenuated with transdermal buprenorphine (20 mcg/hr) as rescue therapy.

**Interpretation:**

This case underscores under-recognized risks of delayed withdrawal during LAIB-facilitated discontinuation and illustrates how loss of kappa opioid receptor (KOR) antagonism may contribute to the emergence or exacerbation of mood-like symptoms (e.g., anhedonia, dysphoria) that can be misattributed to primary psychiatric disorders rather than atypical withdrawal.

**Implications:**

Comprehensive tapering protocols, extended monitoring (6–12 months), reinforcement of recovery capital (defined as the sum of internal and external resources that support sustained recovery), and explicit informed consent about delayed withdrawal are essential when considering LAIB to discontinue buprenorphine. Clinicians should anticipate the need for bridging strategies, including symptomatic agents (such as clonidine or gabapentin), or buprenorphine rescue (temporary or return to maintenance)—and weigh formulation differences (Sublocade vs Brixadi) when planning stepwise tapers.

## Introduction

**Figure 1:**
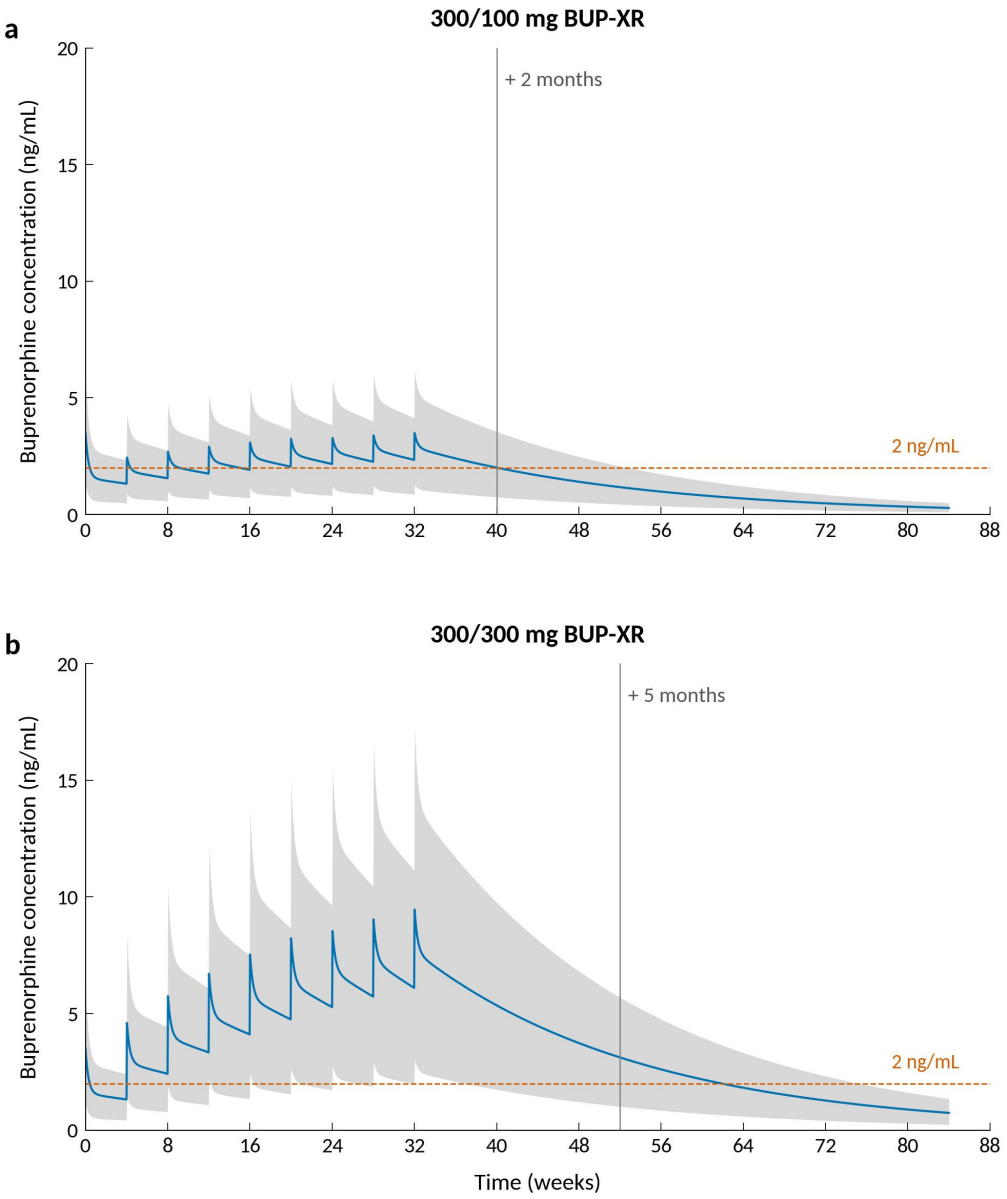
Predicted decrease in buprenorphine plasma concentrations for Sublocade (BUP-XR) dosing regimens following treatment cessation. **(a)** 300/100-mg dosing regimen 2; **(b)** 300/300-mg dosing regimen. Adapted with permission from “Population Pharmacokinetics of a Monthly Buprenorphine Depot Injection for the Treatment of Opioid Use Disorder: A Combined Analysis of Phase II and Phase III Trials,” by Jones et al., 2021 (8), Clinical Pharmacokinetics, 60, pp. 527–540 (https://doi.org/10.1007/s40262-020-00957-0). Copyright 2020 by the Author(s).

Long-acting injectable formulations of buprenorphine (LAIB), including Sublocade and Brixadi, have expanded options for opioid use disorder (OUD) treatment across maintenance and transition phases. Sublocade, in particular, is often viewed as having a “built-in taper,” given its passive prolonged depot-based decline (See **Figure 1**) and the absence of daily dosing, which many clinicians and patients consider a gentler route to discontinuation. Case reports describe successful use of single-injection extended-release buprenorphine to facilitate transition off sublingual therapy (1). Nevertheless, evidence-based guidance on if, when, and how to taper buprenorphine—especially using LAIB—remains sparse.

Understanding LAIB pharmacokinetics is central to evaluating its purported tapering advantages. Sublingual buprenorphine typically has a half-life of ~24–42 hours, whereas Sublocade exhibits an effective half-life of ~43–60 days, with measurable plasma levels persisting for months post-injection (2, 3); in contrast, Brixadi (monthly formulation) has a shorter effective half-life of ~19–26 days, producing a shorter “tail.” These differences shape clinical expectations about the timing and intensity of withdrawal and the feasibility of fine-tuning taper velocity.

Clinically, buprenorphine discontinuation can be challenging and may be perceived by some patients as more difficult than withdrawal from full agonists. Beyond partial μ-agonism, buprenorphine is a potent KOR (kappa opioid receptor) antagonist with antidepressant-like properties, and loss of KOR antagonism during discontinuation may contribute to delayed, atypical symptoms (e.g., emotional blunting, anhedonia, anxiety, fatigue) that do not conform to classic μ-withdrawal physiology (4). These features complicate diagnostic clarity and can be mistakenly treated as primary mood or anxiety disorders if the temporal relation to tapering is missed.

We present a case of delayed withdrawal several months after a single LAIB dose given as a tapering strategy, highlighting pharmacologic, mechanistic, and procedural implications for practice.

## Case presentation

### Patient

Mr. H, a 45-year-old man with OUD in sustained remission (~10 years), hypogonadism, opioid-induced central apnea, and no history of pre-existing mood-related disorders. The patient had long-term stability on 8 mg sublingual buprenorphine twice daily. Acknowledging his strong recovery capital and through shared decision-making, he elected to transition to LAIB as a tapering mechanism. He received a single subcutaneous intra-abdominal 200 mg dose of Sublocade (non-standard dose selected to approximate his daily sublingual exposure). No acute adverse effects occurred.

### Delayed symptoms

Four months post-injection, he developed separate episodes of GI upset, fatigue, malaise, mood-related symptoms, and hyperhidrosis, which were ultimately attributed to emerging withdrawal. The patient reported predominant sleep-related symptoms including nocturnal restlessness, persistent fatigue, and brain fog, alongside increased anhedonia and irritability since symptoms onset.

### Investigations

**Figure 2:**
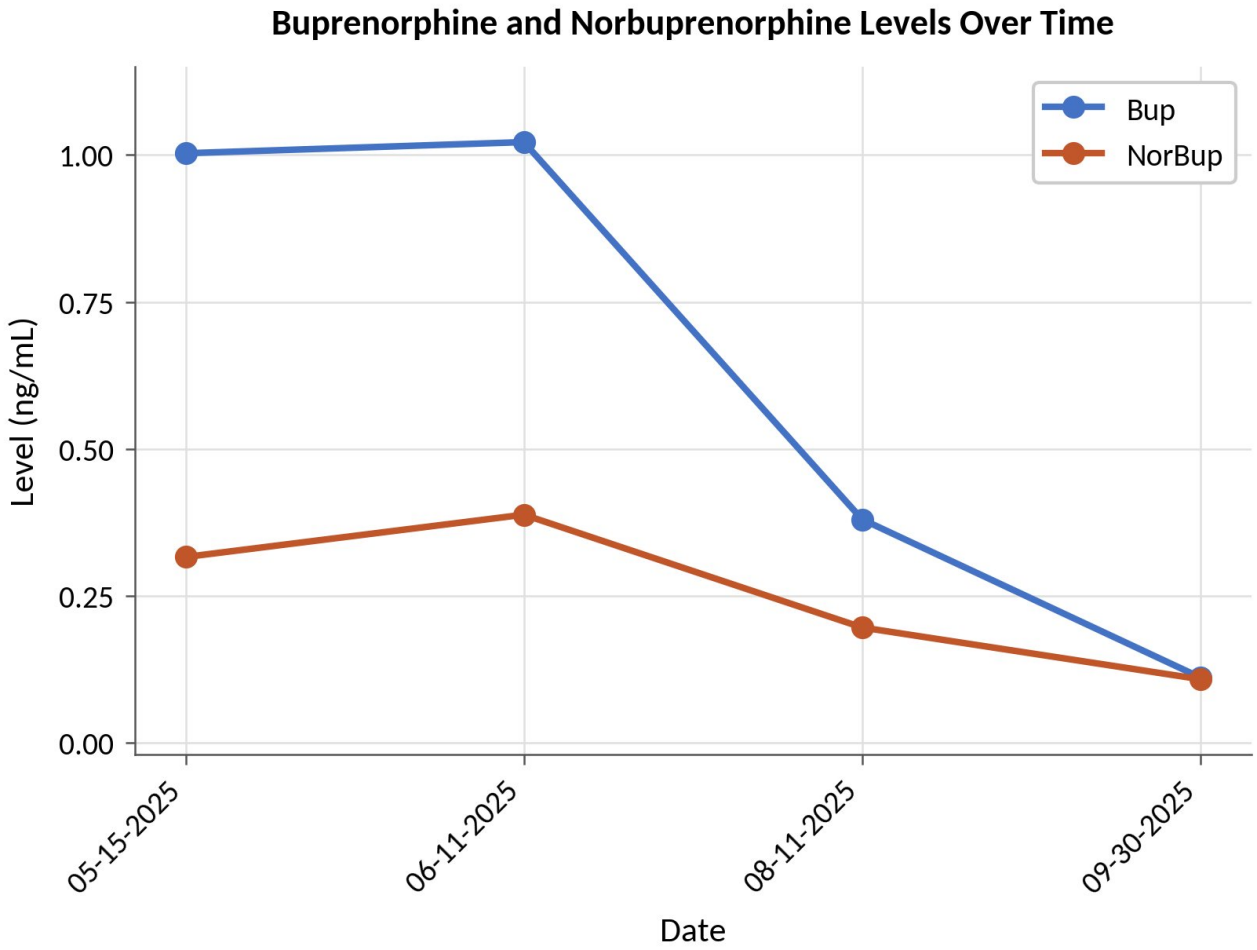
Buprenorphine (BUP) and norbuprenorphine (NorBUP) plasma levels over four sampling points (May–September 2025) following a single 200 mg Sublocade injection, illustrating a gradual decline with symptom onset at ~4 months; note atypical NorBUP < BUP pattern.

Recognizing the known variability of metabolism of LAIB *in vivo*, buprenorphine (BUP) and norbuprenorphine (NorBUP) were followed at four time points across May–September 2025 to inform our clinical decision making. BUP remained high through month 1, declined markedly by month 4, and approached zero by month 5; NorBUP followed a lower, parallel trajectory. The relatively lower NorBUP vs BUP pattern was noted as atypical and merits further exploration. (**Figure 2**).

The diagnostic picture was complicated by the temporal dissociation between the injection and symptom onset, as well as the gradual, non-simultaneous emergence of symptoms. The differential diagnosis therefore included delayed buprenorphine withdrawal, new-onset depressive or anxiety disorder, and an intercurrent viral illness. Delayed buprenorphine withdrawal was favored given the temporal correlation with the declining depot, the multi-system symptom pattern in the absence of any identifiable psychosocial stressor or new psychiatric history. This was further supported by the pharmacokinetic trajectory demonstrating significantly decreased buprenorphine plasma levels coinciding with symptom onset. Administration of naltrexone 12.5 mg at month five served as an inadvertent confirmatory test, precipitating acute worsening of withdrawal and confirming residual μ-opioid receptor occupancy at that time point.

### Management and outcome

At month five, the patient reported partial symptom improvement yet persistent fatigue and brain fog. The clinical picture was attributed to mixed μ-withdrawal and possibly diminishing KOR antagonism as buprenorphine levels fell. A trial of naltrexone was considered to facilitate transition and potentially restore KOR antagonism; after informed consent regarding precipitated withdrawal risks, the patient received 12.5 mg naltrexone (he was offered a micro-induction but chose the higher dose pathway). Two hours later, he developed diarrhea, fatigue, restless legs, and anxiety, consistent with worsening mu opioid withdrawal. Symptoms were mitigated by transdermal buprenorphine (Butrans 20 mcg/hr), with improvement beginning ~6 hours post-placement; the patch was continued for stabilization.

### Clinical timeline

**Figure 3**.

**Figure 3:**
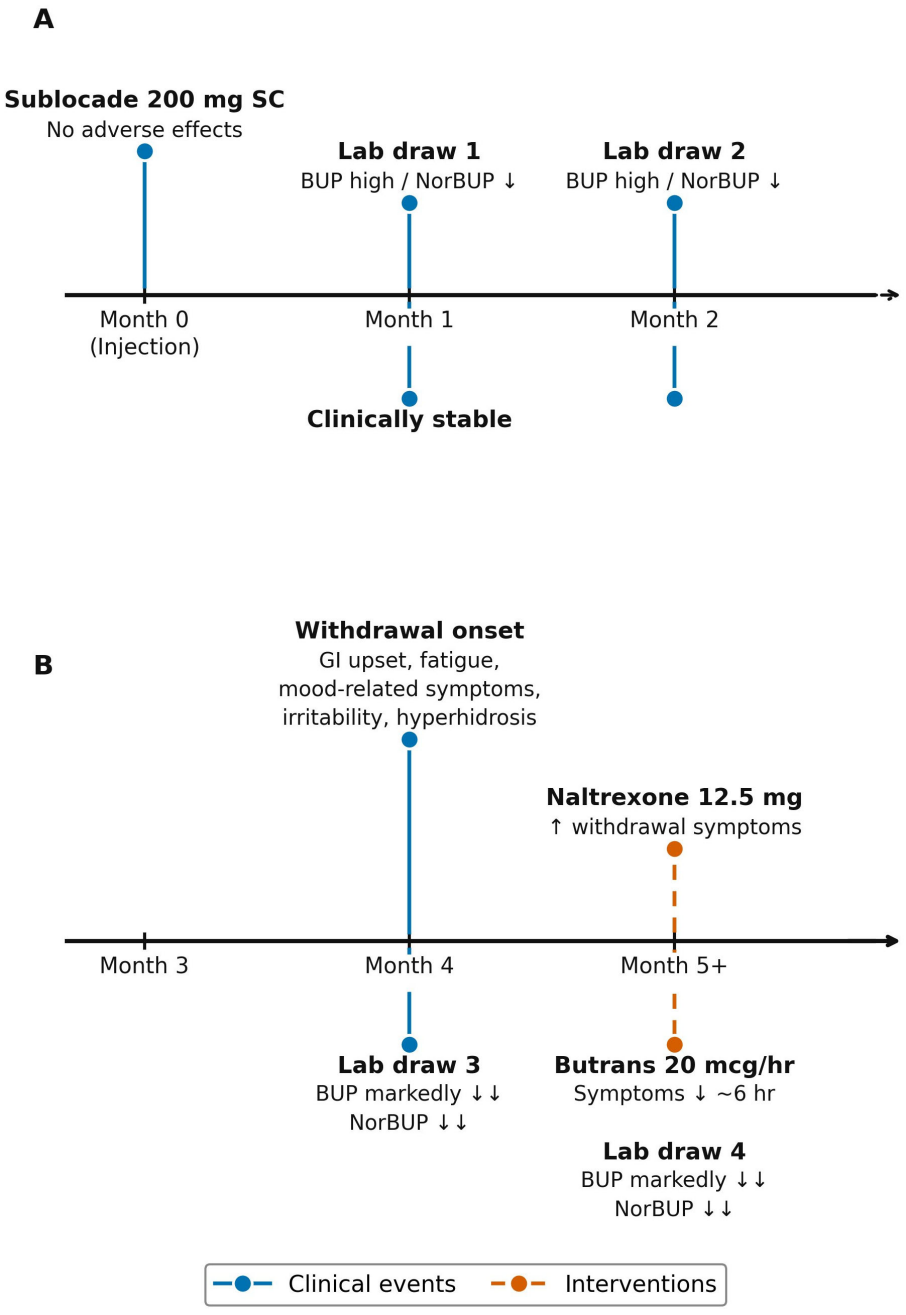
Clinical timeline following a single 200 mg Sublocade injection as a tapering strategy. **(A)** Months 0–2: administration of Sublocade 200 mg SC with no adverse effects, followed by clinical stability and laboratory monitoring (Lab draws 1–2) showing elevated BUP with declining NorBUP. **(B)** Months 3–5+: onset of withdrawal symptoms at month 4 (GI upset, fatigue, mood-related symptoms, irritability, hyperhidrosis) coinciding with markedly decreased BUP/NorBUP levels (Lab draw 3); administration of naltrexone 12.5 mg at month 5 precipitated worsening of withdrawal symptoms, which were subsequently attenuated with transdermal buprenorphine (Butrans 20 mcg/hr; Lab draw 4). Blue markers and solid lines denote clinical events and laboratory findings; orange markers and dashed lines denote pharmacologic interventions.

## Discussion

### Delayed withdrawal with LAIB tapering

This case challenges assumptions that LAIB inherently produces smoother, predictable tapers. Here, classic μ-withdrawal symptoms first emerged at ~4 months, coinciding with declining depot levels, and were exacerbated by naltrexone, indicating persistent receptor occupancy well beyond the injection date. The episode underscores how delayed withdrawal can occur during the depot “tail,” and why symptoms may present after apparent clinical stability.

Buprenorphine withdrawal is often prolonged and variable, rather than acutely intense, and patients frequently report greater difficulty than clinicians anticipate—even with structured tapers and ancillary medications. In controlled settings transitioning to extended-release naltrexone, COWS (Clinical Opiate Withdrawal Scale) scores can remain elevated for weeks, underscoring how symptom burdens persist despite protocolized care (5). While COWS remains a clinically useful tool, its utility may be limited in cases of delayed LAIB withdrawal, where symptoms do not present simultaneously as in classical opioid withdrawal but rather emerge gradually and heterogeneously over weeks to months. As a point-in-time assessment, COWS may fail to capture the full burden of an evolving, atypical withdrawal syndrome, potentially leading to under recognition and undertreatment in this context.

### Role of kappa opioid receptor antagonism

Beyond μ-agonism, buprenorphine’s KOR antagonism could possibly contribute substantially to mood and affective stability. As depot levels wane, loss of KOR antagonism may unmask KOR hypersensitivity, manifesting as dysphoria, anhedonia, anxiety, and fatigue, which clinicians may misinterpret as primary psychiatric symptoms rather than atypical withdrawal. Preclinical and clinical observations support the hypothesis that KOR signaling modulates depressive-like states during abstinence and withdrawal; restoring opioid-system balance (e.g., via antagonism at KOR) may relieve affective symptoms more effectively than serotonergic agents alone (6). In this case, rescue transdermal buprenorphine ameliorated mood-related symptoms, emphasizing the distinct pathophysiology associated with declining KOR blockade.

### Clinical management: education, monitoring, and bridging

Patients often disengage from care during periods of perceived stability, increasing the risk of being lost to follow-up just as delayed withdrawal emerges. Proactive education should normalize the possibility of late-onset symptoms (fatigue, anxiety, irritability, anhedonia) and explain their potential relation to KOR physiology. Extended monitoring 6–12 months after the last LAIB dose may be needed, with readiness to deploy bridging strategies (e.g., α2-agonists, ondansetron, gabapentin, temporary buprenorphine rescue) or re-initiation of buprenorphine if relapse risk or symptom burden becomes clinically significant. Consideration of naltrexone must account for residual depot exposure and the risk of precipitated withdrawal. This extended monitoring window demonstrates a period of physiologic vulnerability, where the risk of disengagement may be elevated. Preserving recovery capital — defined as the internal and external resources (social support, employment, housing, psychological well-being, and treatment engagement) that sustain long-term remission (7) — is the unifying clinical goal when facilitating LAIB discontinuation.

### Limitations

Several limitations warrant consideration. As a single-patient design, generalizability to other LAIB formulations or tapering regimens is inherently limited. Withdrawal severity was not assessed using a standardized instrument such as the Clinical Opiate Withdrawal Scale, limiting objective measures over time. Pharmacokinetic sampling spanned approximately 5 months, and the absence of direct KOR assays limits mechanistic confirmation of KOR-mediated contributions to the delayed withdrawal phenomenon. Together, these limitations highlight a broader clinical gap in literature: standardized protocols for monitoring and characterizing atypical withdrawal during LAIB tapering do not currently exist. We hope this case advances that conversation and encourages further investigation.

## Conclusion

LAIB-facilitated discontinuation can be clinically appealing, but this case demonstrates that delayed withdrawal may occur months after injection, particularly with Sublocade’s longer tail. Without explicit informed consent, extended monitoring, and bridging plans, late-onset symptoms risk misdiagnosis and relapse, especially in patients who disengage from care. We recommend structured taper protocols, education about KOR-related symptomatology, 6–12 months of follow-up after the last LAIB dose, and a low threshold to deploy symptomatic supports or buprenorphine rescue when clinically indicated.

### Learning points (clinical pearls)

1. Delayed withdrawal with LAIB tapering; symptoms may emerge 4–12 months post-injection depending on depot exposure. While naltrexone may help to restore KOR antagonism and address affective symptoms, it must be used with caution given the potential risk of precipitating withdrawal in the setting of residual *µ*-opioid agonism. Gradual induction and informed consent discussing the risk of precipitated withdrawal are required.
2. Loss of KOR antagonism may contribute to affective symptoms (anhedonia, dysphoria) that mimic primary mood disorders—screen for atypical withdrawal.
3. Formulation matters: Sublocade may require at least 12 months of monitoring to safely ensure delayed withdrawal will not be missed (3–6 months after single dose); Brixadi may only require 2–4 months of monitoring.
4. Extend monitoring 6–12 months and encouragement for patients to maintain recovery capital is critical

### Patient perspective

The patient identified discontinuation of buprenorphine as his primary motivation for transitioning to LAIB. He reported feeling adequately informed prior to the transition regarding the possibility of delayed withdrawal symptoms emerging weeks to months after the final injection. At peak severity, he rated his withdrawal symptoms as 5 out of 10 on a numerical rating scale. He reported feeling well-supported by his care team throughout the taper period and did not disengage from care at any point during or after discontinuation.

The patient reported strong recovery capital during the taper period, attributing this in part to his professional engagement in recovery-oriented programs and active participation in community support groups. In retrospect, he expressed that he would have benefited from more extensive preparation and anticipatory guidance regarding the possibility of withdrawal symptoms emerging several months after his last injection.

## Data Availability

The raw data supporting the conclusions of this article will be made available by the authors, without undue reservation.
